# Percutaneous Endoscopic Colostomy: A New Technique for the Treatment of Recurrent Sigmoid Volvulus

**DOI:** 10.4103/1319-3767.80391

**Published:** 2011

**Authors:** Vipul D. Yagnik

**Affiliations:** Ronak Endo-Laparoscopy and General Surgical Hospital, Patan, Gujarat, India E-mail: vipul.yagnik@gmail.com

Sir,

I read the article entitled, “Percutaneous endoscopic colostomy: A new technique for the treatment of recurrent sigmoid volvulus,”[1] with great interest. I would like to congratulate the author for an excellent description of the technique. However, certain aspects need clarification. Author states that all previously reported series or case reports used a single percutaneous endoscopic colostomy (PEC) tube of small size.[1] Choi *et al*[2] described fixation of the sigmoid colon at two points, one distal and the other proximal to the sigmoid apex with the help of a PEG kit. In large series, Baraza *et al*.[3] had also performed the procedure with similar technique using a 20-F PEG kit. PEC is an alternative to surgery in high-risk/selected patients with recurrent sigmoid volvulus, refractory colonic pseudo-obstruction, or severe slow-transit constipation/fecal constipation. It is important to note that PEC is not recommended for acute sigmoid volvulus or acute colonic pseudo-obstruction, for which the mainstay of treatment is endoscopic or pharmacological decompression.[4] Although placement of the PEC tube is a relatively safe and easy procedure[1], it is not without complication. Other complications in addition to peritonitis are:[3] Major complications (tube migration, recurrence, death), minor complications (abdominal wall bleed, buried bumper, infection, pain, urgency), failure, and poor tolerance. Author statement about the longest follow-up of his case[1] also needs clarification. The longest reported follow-up in literature is 89 months.[3]
